# Staying Connected during the COVID-19 Pandemic: Experiences of Older People in Mexico and Scotland

**DOI:** 10.1093/geroni/igab046.3527

**Published:** 2021-12-17

**Authors:** Louise McCabe, Verónica Montes de Oca, Nereide Curreri, Marissa Vivaldo, Alison Dawson, Miguel Rivera, Maria Arroyo, Elaine Douglas

**Affiliations:** 1 University of Stirling, Stirling, Scotland, United Kingdom; 2 National Autonomous University of Mexico (UNAM), National Autonomous University of Mexico (UNAM), Distrito Federal, Mexico; 3 North-West University, South Africa, Vanderbijlpark, North-West, South Africa; 4 National Autonomous University of Mexico, Mexico City, Distrito Federal, Mexico; 5 Universidad Juarez del Estado de Durango, Universidad Juarez del Estado de Durango, Durango, Mexico

## Abstract

During the pandemic older people saw transformations in their social connections due to lockdowns and other restrictions. Technology provided one mechanism for them to stay connected with others, but technology may not be accessible or desirable for everyone. Gender, socioeconomic status, ethnicity, age and other factors enhance or limit engagement with technology. This project explored experiences of older people in Mexico and Scotland during the pandemic and examined the potential of everyday technology to help maintain social connectedness. A mixed methods approach included secondary analysis of large-scale datasets alongside primary data. Online semi-structured interviews and focus groups were carried out with 36 older people in Mexico and 23 older people in Scotland. Sampling was purposeful creating a diverse sample across age, gender, ethnicity and socioeconomic status. The findings demonstrate that advantages and disadvantages accumulated in the life course determine how older people select, optimize and compensate for new ways of staying socially connected during the pandemic in both countries. The use of technologies among older people is further mediated by structural inequalities with differences found between Mexico and Scotland in specific patterns identified. Further, stereotypes about older age and technology use are obstacles to the use of technology, as they affect the perception of self-efficacy by older people. Despite the obstacles, this study has shown that older people have a broad range of resources that have enabled them to cope with the pandemic and utilise technology to maintain social connections. The project offers recommendations to support older people’s human rights.

